# Pharmacokinetic background of therapeutic drug monitoring in this case

**DOI:** 10.1192/j.eurpsy.2025.227

**Published:** 2025-08-26

**Authors:** M. Stuhec

**Affiliations:** 1 Pharmacology Department, Medical Faculty Maribor, University of Maribor, Maribor; 2 Clinical Pharmacy, Ormoz Psychiatric Hospital, Ormoz, Slovenia

## Abstract

**Abstract:**

The pharmacological effect is the result of both pharmacokinetic (e.g., how the body affects the medication) and pharmacodynamic (e.g., how the medication affects the body) processes occurring simultaneously. Pharmacokinetics includes the processes of liberation, absorption, distribution, metabolism, and elimination (LADME). These processes govern the steady-state plasma concentration (Css). Therapeutic drug monitoring (TDM) is a pharmacological tool used to select and adjust medications. TDM enables Css measurements and dose adjustment calculations. In this presentation, the presenter will cover the main pharmacokinetic issues related to TDM in this clinical case, including Css determination, therapeutic range, dose adjustment, and pharmacokinetic calculations. Participants will learn the fundamental pharmacokinetic aspects necessary for understanding the case.

**Disclosure of Interest:**

None Declared

